# Rhizosphere Microbiome of Arid Land Medicinal Plants and Extra Cellular Enzymes Contribute to Their Abundance

**DOI:** 10.3390/microorganisms8020213

**Published:** 2020-02-05

**Authors:** Abdul Latif Khan, Sajjad Asaf, Raeid M. M. Abed, Yen Ning Chai, Ahmed N. Al-Rawahi, Tapan Kumar Mohanta, Ahmed Al-Rawahi, Daniel P. Schachtman, Ahmed Al-Harrasi

**Affiliations:** 1 Natural and Medical Sciences Research Centre, University of Nizwa, Nizwa 616, Sultanate of Oman; sajadasif2000@gmail.com (S.A.); ahmed.alrawahi@unizwa.edu.om (A.N.A.-R.); nostoc.tapan@gmail.com (T.K.M.); ahmed@unizwa.edu.om (A.A.-R.); 2 Sultan Qaboos University, College of Science, Biology Department, Muscat 123, Sultanate of Oman; rabed@squ.edu.om; 3 Department of Agronomy and Horticulture and Centre for Plant Science Innovation, University of Nebraska-Lincoln, Lincoln, NE 68588, USA; Yenning_hala@hotmail.com (Y.N.C.); daniel.schachtman@unl.edu (D.P.S.)

**Keywords:** arid land, medicinal plants, microbiome, diversity, metagenomics, extra cellular enzymes

## Abstract

Revealing the unexplored rhizosphere microbiome of plants in arid environments can help in understanding their interactions between microbial communities and plants during harsh growth conditions. Here, we report the first investigation of rhizospheric fungal and bacterial communities of *Adenium obesum*, *Aloe dhufarensis* and *Cleome austroarabica* using next-generation sequencing approaches. *A. obesum* and *A. dhufarensis* grows in dry tropical and *C. austroarabica* in arid conditions of Arabian Peninsula. The results indicated the presence of 121 fungal and 3662 bacterial operational taxonomic units (OTUs) whilst microbial diversity was significantly high in the rhizosphere of *A. obesum* and *A. dhufarensis* and low in *C. austroarabica.* Among fungal phyla, *Ascomycota* and *Basidiomycota* were abundantly associated within rhizospheres of all three plants. However, *Mucoromycota* was only present in the rhizospheres of *A. obesum* and *A. dhufarensis*, suggesting a variation in fungal niche on the basis of host and soil types. In case of bacterial communities, *Actinobacteria*, *Proteobacteria*, *Bacteroidetes*, *Planctomycetes*, *Acidobacteria,* and *Verrucomicrobia* were predominant microbial phyla. These results demonstrated varying abundances of microbial structure across different hosts and locations in arid environments. Rhizosphere’s extracellular enzymes analysis revealed varying quantities, where, glucosidase, cellulase, esterase, and 1-aminocyclopropane-1-carboxylate deaminase were significantly higher in the rhizosphere of *A. dhufarensis,* while phosphatase and indole-acetic acid were highest in the rhizosphere of *A. obesum*. In conclusion, current findings usher for the first time the core microbial communities in the rhizospheric regions of three arid plants that vary greatly with location, host and soil conditions, and suggest the presence of extracellular enzymes could help in maintaining plant growth during the harsh environmental conditions.

## 1. Introduction

The arid or semi-arid land covers almost 26% of the earth’s ecosystems, where life is constrained and often confronted with extremely low water and high temperature. The vegetation is either succulent (accumulating water) or non-succulent perennial hard plants. Both are true xerophytes and are well adapted to the low water conditions [1]. However, in such harsh climatic conditions, endemic micro-symbionts are of great importance for plant survival [2]. Understanding the role of microbial communities and their association with plants during their growth, development, and extreme conditions in arid environments are of considerable interest to ecologists [3,4,5]. The microorganisms that are predominantly present in the rhizosphere have been shown to play a role in the transport of mineral nutrients, secretion of secondary metabolites, and mitigation of abiotic and biotic stresses [6,7,8,9,10]. During microbial association with the host plants, bacteria and fungi produce various extracellular enzymes that convert the macromolecules into transportable simpler products that can be distributed throughout the plant cells [11,12,13]. In addition to the initiation of the host-symbiosis process, some of these exozymes hinder the plant pathogenic infections and boost abiotic stress tolerance [14,15]. The plant, on the other hand, facilitates a suitable niche for distinct microbes to grow and reproduce while mutually sharing beneficial exudates and nutrients [16,17]. Such interactions between the microbial communities and medicinal plants have been minimally investigated, particularly in arid ecosystems [5]. Previous studies [18,19,20,21,22,23] have evaluated the microbiome, especially the bacterial communities from arid soil; however, no studies have been performed on the rhizosphere microbiomes of arid plants.

Despite the importance of the plant life in the arid environments, little is known about their associated endemic microflora [17]. Recently, some studies have been performed on the rhizospheric bacterial microbiomes of plants growing in the arid land ecosystems [24,25,26,27]. The analyses of microbiomes of various cultivated plants, including *Agave species, Zea mays, Phaseolus vulgaris, Ainsliaea henryi* Diels, *Dioscorea opposita*, *Potentilla discolor* Bge, *Stellera chamaejasme* L., *Ophiopogon japonicus* (Thunb) Ker-Gawl., *Juncus effusus* L. var. *decipiens* Buchen., and *Rhizoma arisaematis* [28,29] showed remarkably high and diverse rhizosphere colonization with *Actinobacteria* [30]. In addition, some of the recent studies have elucidated the rhizosphere communities of *Rehmannia glutinosa* [31], *Rumex patientia* [32], *Polygonum cuspidatum* [33], *Aloe vera* [34], *Rhododendron arboretum* [35], and *Thymus zygis* [36]. These studies have been restricted to the bacterial communities and did not include fungi, and a few studies used high-throughput next-generation sequencing. However, the importance of understanding the microbiome composition of wild plants growing in the arid environments has at least been demonstrated till now.

**Figure 1:**
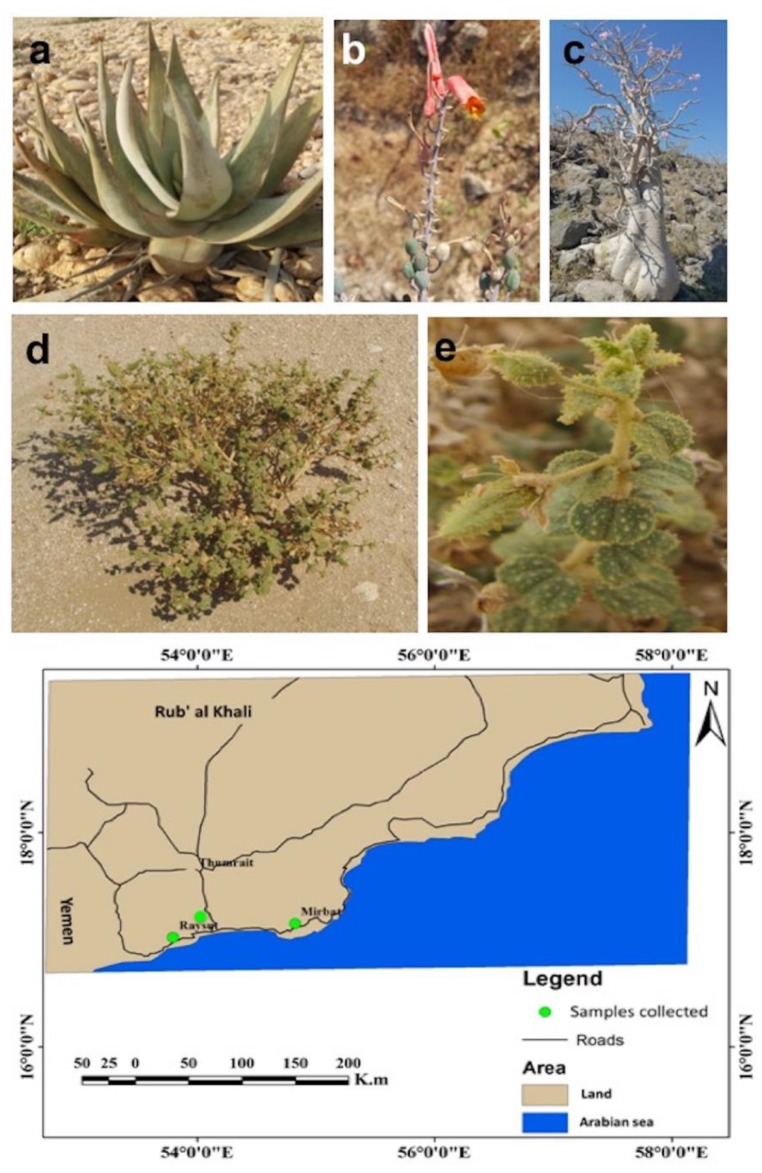
Plant habitats and their location. (**a**) *A. dhufarensis* growing wildly and (**b**) its flowering part; (**c**) *A. obesum* plant and (**d**,**e**) *C. austroarabica* habitat and phyllosphere part. All rhizosphere samples were collected from three different locations. The map for sample collection was made in ArcGIS v9.3.1 (Redlands, CA, USA; http://www.esri.com/software/arcgis/eval-help/arcgis-931).

In the present study, we have investigated the microbiomes of three plants (*A. dhufarensis, C. austroarabica,* and *A. obesum*) collected from different areas of the arid land that have previously not been explored. *A. obesum* and *A. dhufarensis* are more concentrated in the tropical arid environments, whereas *C. austroarabica* inhabits in extremely arid environments (Figure 1). Moreover, these plants are ecologically and medicinally important too. The plants growing in such an environment often experience a wide array of environmental stresses, including UV irradiation, high heat, drought and strong wind. Rainfall in this region is very limited (<80 mm per annum) and occurs for very short periods. *A. dhufarensis*, an endemic plant to the Dhofar region in Oman [37], is the least studied but has shown to possess antioxidant potentials [38]. The crushed leaves of *C. austroarabica* produce fragrance. *A. obesum* is often known as arid rose, and local people use it to treat wounds, venereal diseases, skin diseases, tooth decay, headaches, and muscle pain [39]. These three species are the representative plants in the arid lands of Oman and the Arabian Peninsula and are often exposed to the harsh environmental conditions. However, despite the exposure to the high drought, heat and strong UV conditions, these plants survive for long periods of time. Herein, we investigated for the first time the fungal and bacterial communities associated with the rhizosphere of these three plants species. Comparative studies across the microbiomes allowed us to explore the major and prominent microbial players in the arid plant life.

## 2. Methods

### 2.1. Study Site

The soil samples from the rhizosphere of *A. obesum* (AO; 17°01′11″ N 54°08′23″ E)*, A. dhufarensis* (AD; 17°01′11″ N 54°05′23″ E) and *C. austroarabica* (CA; N19°29.47′ E054°49.81) were collected from the Dhofar region of the Sultanate, Oman during the dry summer season (June 2016). The rhizosphere soils adjacent to the root surface (5 to 60 inches deep) were collected. For each plant species, thirty soil samples of root rhizosphere regions were collected, which were later pooled into three replicates (ten plants in each replicate). The replicates were approximately 500 m apart from each other. In contrast, each plant species was 30 to 50 km apart from each other (Figure 1). The climate of the area is dry and predominant features of dry tropical to arid land weather conditions are prevailing. To understand the soil physical and chemical properties, detailed soil chemical analysis was performed according to the method of Adhikari et al. [40].

### 2.2. DNA Extraction and MiSeq Sequencing

After pooling the soil samples, the mixtures of each rhizosphere soil from each plant species (100 g, in triplicate) were mixed and subjected to the total DNA extraction using the MoBio Power Soil DNA Extraction Kit. PCR free libraries of each DNA sample were generated by amplifying the internal transcribed spacer (ITS2 and ITS4) and 16S rRNA (V3-V4) for fungal and bacterial communities, respectively. For 16S rRNA, peptide nucleic acid (PNA) clamps were used to reduce the mitochondrial and chloroplast contamination. A paired-end sequencing approach of 250 bp was conducted on an Illumina MiSeq instrument (Illumina Inc., San Diego, CA, USA) operating with v2 chemistry (User Guide Part # 15,027,617 Rev. L). All quality reads related to the study are available at NCBI under BioProject PRJNA337739, 16S Accessions (KDUM00000000, KDUL00000000, KDUK00000000), and ITS Accessions (KDUJ00000000, KDUI00000000, KDUH00000000).

### 2.3. Data Processing and Analyses

**Figure 2:**
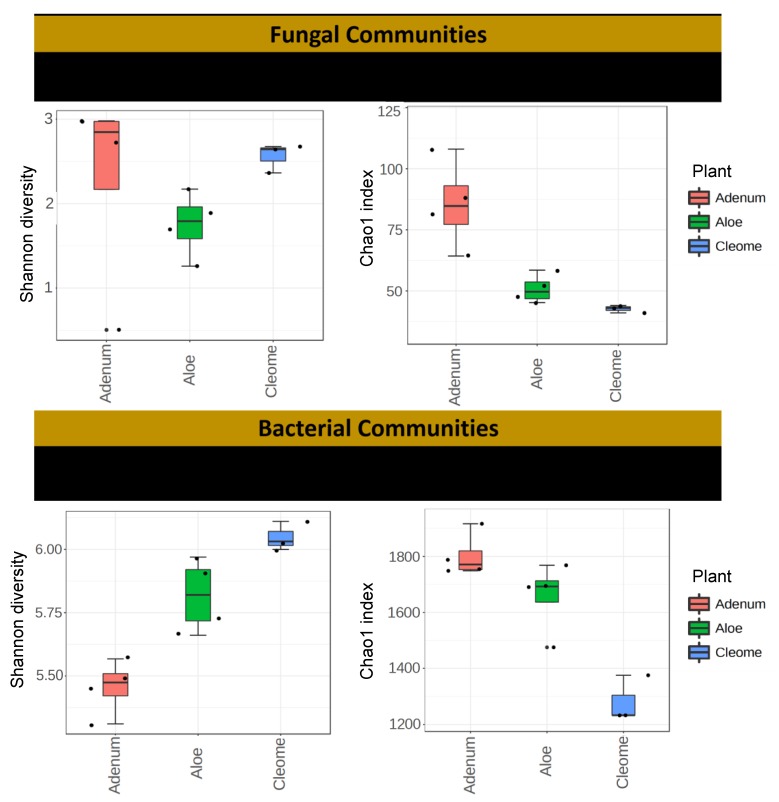
Distribution of operational taxonomic units (OTUs) for fungal and bacterial communities and Chao-1 of each replica from data generated through MiSeq sequencing (16S and ITS) of the rhizosphere samples from *A. dhufarensis, A. obesum* and *C. austroarabica*. Nonmetric multidimensional scaling (NMDS) plots for Bray–Curtis distances of fungal and bacterial communities associated with the three plant species. Shannon index is presented in the left and Chao1 is presented in the right side of the figure.

The raw sequence reads were merged, trimmed, filtered, and clustered at 97% identity for fungal and bacterial sequences using the UPARSE pipeline [41]. Taxonomies were assigned to each bacterial OTU using the RDP Naïve Bayesian Classifier [42] trained on the Greengenes database [43]. Fungal taxonomy assignment was performed with the Naïve Bayer classifier trained on the UNITE reference database [42]. The OTUs whose taxonomic classifications did not match to their expected kingdoms (bacteria and fungi) were removed. Each sample was rarefied to 96,961 and 26,999 for bacterial and fungal reads, respectively, prior to alpha and beta diversity analysis. For the alpha diversity, the Chao1 index and Shannon diversity were used to determine the species richness and diversity in the samples (Figure 2). The plots for Chao1 index and Shannon diversity were generated with Microbiome Analyst [44]. For beta diversity, canonical analysis of principal coordinate (CAP) was performed based on Bray-Curtis and both weighted and unweighted UniFrac distances using the Vegan package in R (Version 1.0.44) [45]. Canonical correlation analysis (CCA) was employed to examine the relationship between the microbes and measured enzyme activities [46].

### 2.4. Microbial Products in Rhizosphere Soils

To estimate the extracellular enzymes (glucosidase, phosphatase, esterase, and cellulase), the method reported by Marx et al. [47] and Khan et al., [15] was used with minor modifications. Briefly, all chemical reagents were obtained from Sigma-Aldrich Co. Ltd. (Munich, Germany). A 10 mL aliquot of a 10 mM stock solution of each 4-methylumbelliferone (MUB) substrate was prepared. The assay procedures for all the substrates were the same. A 7-MUB standard was used for the study. A stock solution of MUB (10 mM) was prepared in methanol (0.1762 g of 4-methylumbelliferone in 100 mL) that was subsequently diluted to 1 µM in sodium acetate (pH 5.2) buffer. The soil samples were analyzed for exozymes as described by Marx et al. (2001) [47] using a fluorescence spectrophotometer (Shimadzo, Tokyo, Japan). The methodology of Honma and Shimomura was applied for ACC deaminase activity with some modifications as described by Shaharoona et al. [48], and the amount of α-ketobutyrate produced from the hydrolysis of ACC was measured (Supplementary methods). The quantification of indole-3-acetic acid in the soil sample was performed as described by Khan et al. (2016) [15].

### 2.5. Statistical Analysis

At least three replicate samples were analyzed during this study. The data for the enzyme study are presented as the mean ± standard error of the mean (SEM). The significant differences were studied using ANOVA (one-way analysis of variance) approach. The significant differences were considered significant at *p* < 0.05 and were calculated by GraphPad Prism Version 6.01 (GraphPad Software, San Diego, CA, USA). Duncan’s multiple range test at *p* < 0.05 (SAS 9.1, Cary, NC, USA) was used to compare the mean values. For the multivariate analyses, statistical analysis was performed using permutational multivariate analysis of variance (PERMANOVA) with 999 permutations.

## 3. Results

### 3.1. Soil Variations among Three RHIZOSPHERES

The soil analysis of the three rhizosphere regions of *A. dhufarensis*, *A. obesum* and *C. austroarabica* showed variability among two locations (dry tropical to arid). The two plants from arid land (*A. obesum* and *A. dhufarensis*) showed a similar pattern (*p* < 0.05; insignificant statistically) of distribution of various physical (temperature, moisture content, clay, sand, silt, gravel, bulk density, organic matter, and texture) and chemical (electrical conductivity, pH, nitrates, and phosphorus) soil quality parameters as compared to *C. austroarabica* suggesting an extreme growth conditions (Table 1).

### 3.2. Microbial Diversity in the Rhizosphere of the Three Plants

A total of 361.4 Mb and 0.786 Gb of high-quality read data (Q20% 98.64; Q30% 94.31) were generated for fungal and bacterial communities, respectively (Table S1). The mean read count was 110,712 ± 13.87 and 194,927 ± 11.32 for ITS and 16S, respectively (Table 2; Figure S1). In the case of fungi, the highest average read count was obtained in *A. dhufarensis* (13,0150 ± 38.81). A total average of 102,062 ± 16.31 and 99,923 ± 18.89 fungal reads were generated from *A. obesum* and *C. austroarabica*, respectively. In the bacterial community analysis, the highest average read count was identified in *C. austroarabica* (200,875 ± 14.73), followed by *A. obesum* (197,910 ± 9.01). *A. dhufarensis* had the lowest average read count (185,997 ± 16.76; Table 2).

The number of OTUs calculated using the subsets with the same number of sequences for the three plant samples ranged from 50 to 229 for fungal communities and from 2812 to 4834 for bacterial communities (Table 1; Table S2). In overall comparison among microbial communities, the current result suggests a higher abundance of bacterial communities than fungal communities. Fungal OTU richness and diversity were significantly highest (*p* < 0.076) in *A. obesum* (201 ± 1.3) and lowest in *C. austroarabica* (54 ± 1.9; Figure 2; Table 2). This was further validated with Chao1 analysis showing rare OTUs in the three replicates of each sample (Figure 2; Table 2). The abundance-based coverage showed a significantly higher (*p* < 0.001) fungal diversity in the rhizosphere of *A. obesum* than the other two plants. In the case of bacterial communities, the significantly highest (*p* < 0.0014) number of OTUs was obtained from *A. dhufarensis* (4014 ± 33.6) and the lowest number of OTUs was obtained from *C. austroarabica* (3427 ± 5.3) (Table 2). Similarly, Chao 1 was significantly higher (*p* < 0.0034; 1764 ± 37.2) in *A. obesum* compared to *A. dhufarensis* (1718 ± 6.2) and *C. austroarabica* (1280 ± 8.6; Table 2). This shows the presence of higher abundance of individual species in *A. obesum* that reflects the singleton of species richness in fungi and bacteria. In the case of Shannon diversity indices, there was a non-significant difference in the fungal diversity of *C. austroarabica* and *A. obesum*, and the Shannon diversity index was significantly lower in *A. dhufarensis*. In contrast, the bacterial population was significantly higher (*p* < 0.019; 5.8 ± 3.1) in *C. austroarabica* than *A. obesum* (5.4 ± 0.7) and *C. austroarabica* (6.3 ± 2.6; Table 2; Table S3).

**Figure 3:**
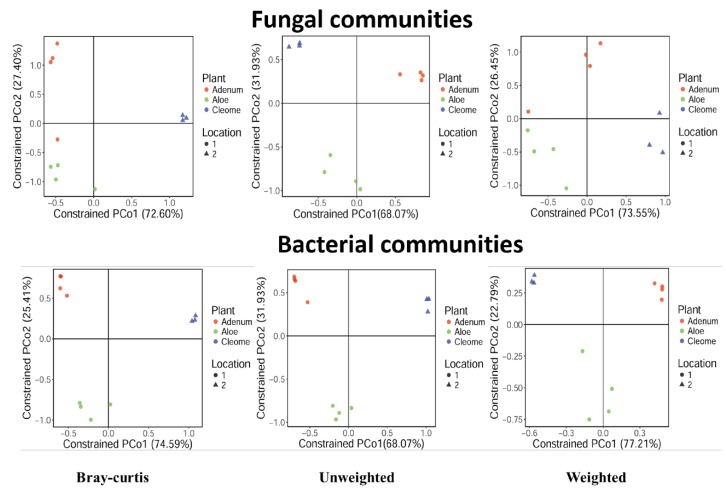
Constrained analysis of principal coordinates (CAP) plots for the fungal and bacterial communities in the rhizosphere of *A. dhufarensis, A. obesum,* and *C. austroarabica*. The communities were constrained by geographical location (1 –sub-tropical arid conditions and 2 – absolute arid conditions). The CAP analysis was performed on Bray–Curtis, weighted and unweighted distances. The significance of the CAP models was evaluated using ANOVA with 999 permutations.

The abundance of 16S sequences with regards to the location 1 (dry tropical) and 2 (arid) was analyzed using Bray-Curtis, weighted, and unweighted principal component analysis. The Bray-Curtis analysis of fungal communities showed that *A. dhufarensis* and *C. austroarabica* showed their presence in both the locations. However, *A. obesum* showed its partial presence in both the locations (Figure 3). Similarly, for bacterial communities, *A. dhufarensis* was found in both locations, whereas *C. austroarabica* was found to a lesser extent (Figure 3; Figure S2).

### 3.3. Rhizosphere Fungal Diversity

**Figure 4:**
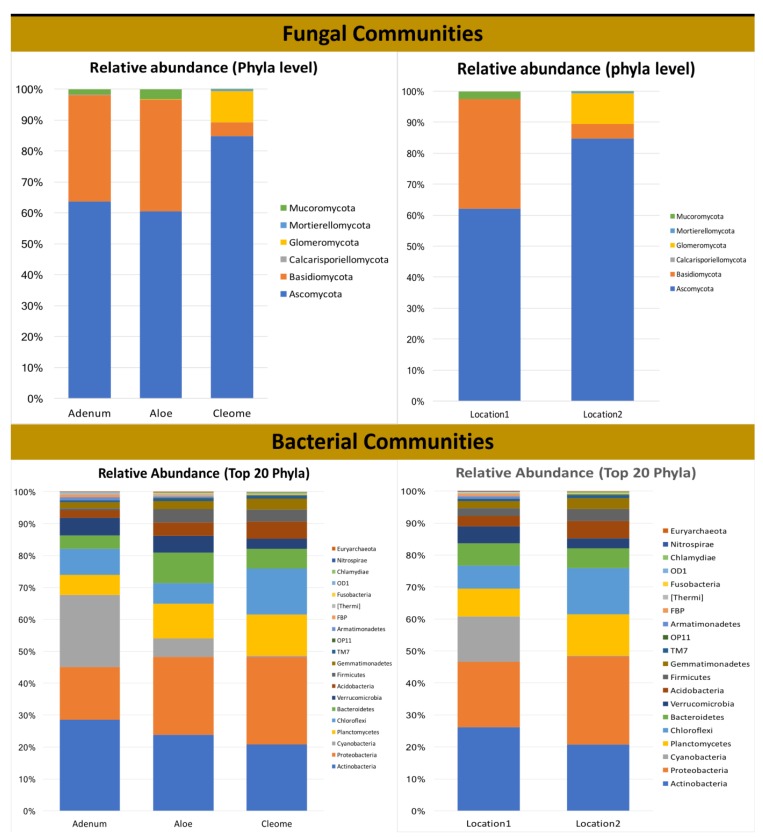
Relative abundances and shared core OTUs of fungal and bacterial phyla and the genera found in the rhizosphere of *A. dhufarensis, A. obesum* and *C. austroarabica*.

Unidentified fungal sequences constituted ~9% to 42.5% of the total sequences of the three plants. These unidentified sequences were significantly higher (68.46%; *p* < 0.0001) in *C. austroarabica* than in the other two plants. Two major phyla, *Ascomycota* and *Basidiomycota,* were detected in all of the studied plant species (Figure 4). However, Mucoromycota was only present in the *A. obesum* and *A. dhufarensis* rhizospheres, suggesting a variation in the fungal niche. In addition, *C. austroarabica* is from an extremely arid environment (location 2), and the presence of Glomeromycota and Mortirellomycota is an interesting feature of its rhizosphere. Furthermore, the abundance of Basidiomycota appreciably reduced and Glomeromycota increased in *C. austroarabica* compared to *A. obesum* and *A. dhufarensis*, indicating an influence of location-specific changes in the microbial population. *Ascomycota* was the most predominant (34.97%) phylum in the rhizosphere samples of the three plants. Its relative abundance was significantly highest in *C. austroarabica* (72.90%; *p* < 0.001) and lowest in *A. dhufarensis*. *Basidiomycota* was the second most dominant phylum and was found to be significantly higher in *A. dhufarensis* (14.53%) than in *A. obesum* (13.8%) and *C. austroarabica* (3.1%) (Figure 4; Table S4).

**Figure 5:**
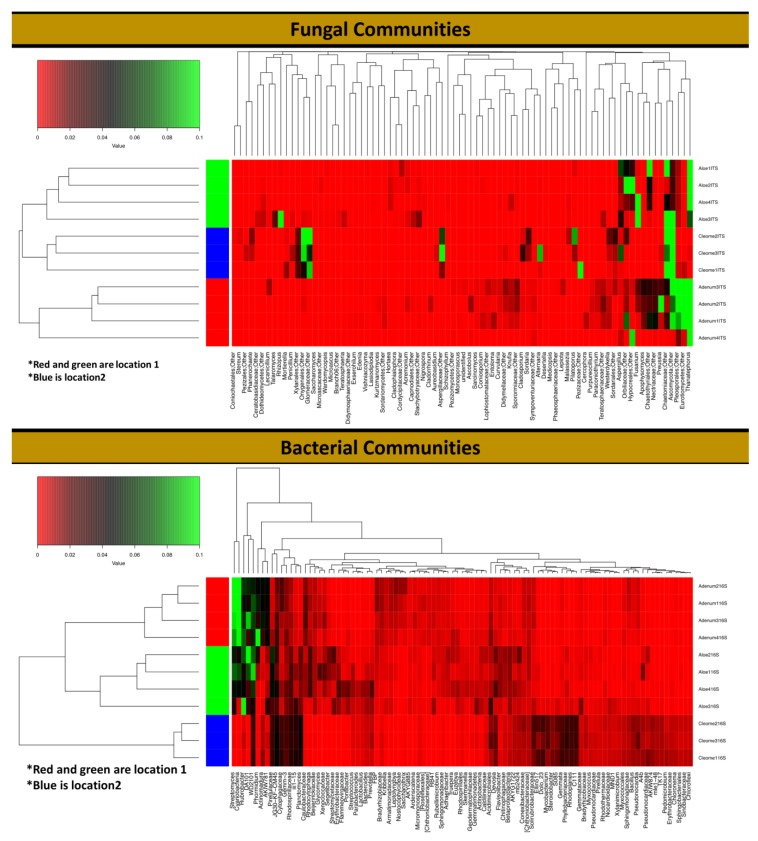
Heat map and dendrogram generated for fungal and bacterial communities in the rhizosphere of *A. dhufarensis, A. obesum* and *C. austroarabica* using highly abundant OTUs across all of the samples and their replicates.

Different genera were encountered in the three-plant species, with *Acremonium* as the most abundant in *A. obesum* (9.17%) and *A. dhufarensis* (4.92%) compared to *C. austroarabica*. *Ascotricha* was only abundant in *A. dhufarensis* (14.77%). Similarly, *Ceratobasidium* (~6.7%) was highly abundant in *A. obesum* (13.93%) and *A. dhufarensis* (12.95%). In contrast, *Corynascus* (5.63%) was abundant in *C. austroarabica*. *Aspergillus, Paecilomyces, Preussia, Alternaria,* and *Teratosphaeria* were also among the abundant genera (Figure 4). Cluster analysis with regard to sample collection (site 1 and site 2) showed that *A. dhufarensis* and *C. austroarabica* grouped together for their abundance in fungal communities (Figure 4). *Rhizopus, Orbiliaceae, Hypocreales,* and *Fusarium* were only specific to *A. dhufarensis,* whereas Ascomycota, Chaetomiaceae, Eurotiomycetes, and Thanatephorus were abundant in both the locations (Figure 4 and Figure 5). However, *Pleosporales* was specific to the location 2 and only found in *A. obesum*. The location-specific (location 1 and location 2) diversity was studied for the fungal communities using Bray-Curtis and unweighted and weighted analyses. The Pco1 for the Bray-Curtis, unweighted, and weighted analyses were 72.6%, 68.07%, and 73.55%, respectively, whereas Pco2 for the Bray-Curtis, unweighted, and weighted analysis were 27.4%, 31.93%, and 26.45%, respectively (Figure 3). The weighted study measures the number of species within the population represented by each member of the sample, whereas in the unweighted, all samples were weighted equally.

### 3.4. Rhizosphere Bacterial Diversity

A total of 32 bacterial phyla were detected, including *Actinobacteria* (26.43%), *Proteobacteria* (17.55%), *Cyanobacteria* (10.9%%), *Planctomycetes* (7.57%), and *Verrucomicrobia* (7.68%). The relative abundance of *Actinobacteria* (31.21%; *p* < 0.0002) and *Cyanobacteria* (26.88%; *p* < 0.0032) was higher in *A. obesum* than *A. dhufarensis* and *C. austroarabica* (Figure 4 and Figure 5). *Bacteroidetes* (12.74%) were higher in *A. dhufarensis,* whereas *Chloroflexi* (10.34%) and *Acidobacteria* (5.48%) were abundant in *C. austroarabica* (Figure 4; Table S5). In total, 274 different genera were found in all the plants, although 34 of the total sequences could not be assigned to known the genera (Figure 4). *Firmicutes* were present in *A. dhufarensis* and *C. austroarabica* but was absent in *A. obesum* (Figure 4). *Euryarchaeota* was found in *A. obesum* and *A. dhufarensis*, whereas it was absent in *C. austroarabica* (Figure 5). Overall, *Streptomyces* (5.81%), *Actinomadura* (1.62%), *Rubrobacter* (6.67%), *Ohtaekwangia* (1.34%), *Bacteroides* (0.75%), *Sphaerobacter* (1.99%), *Gemmatimonas* (1.17%), *Pirellula* (0.90%), *Planctomyces* (1.54%), *Microvirga* (1.66%), and *Sphingomonas* (2.31%; Figure 4) were the most abundant across the three medicinal plants. Across the rhizosphere of the three plants, sequences belonging to the genera *Streptomyces*, *Rubrobacter*, *Spartobacteria,* and *Sphingomonas* were significantly abundant in *A. dhufarensis* and *A. obesum* compared to *C. austroarabica*. However, the genera *Ilumatobacter, Mesorhizobium, Ohtaekwangia, Solirubrobacter, Pirellula, Planctomyces,* and *Sphaerobacter* were abundantly distributed in *C. austroarabica*. A location-specific study of bacterial communities revealed quite universal abundance, except for a few cases. In location 1, *Streptomyces* species were found in *A. obesum,* whereas Rubrobacter was found in *A. obesum* and *A. dhufarensis* (Figure 5; Table S6). In location 2, the abundance level of Proteobacteria was higher than the location 1.

### 3.5. Exozymes, ACC Deaminase and IAA in the Rhizosphere

**Figure 6:**
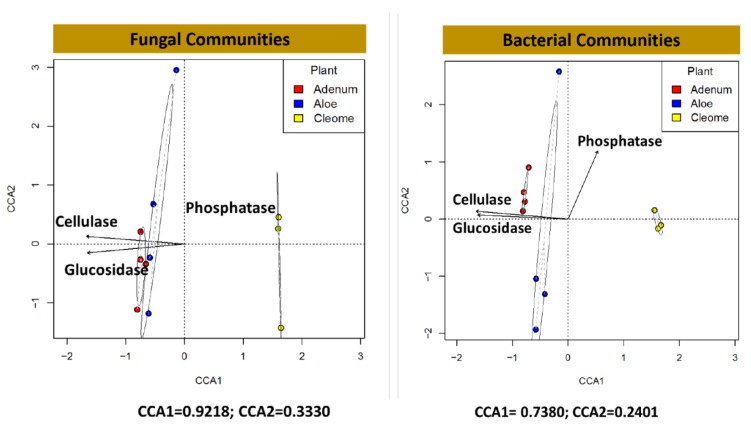
Correspondence analysis (CCA) for fungal and bacterial communities in the rhizosphere of *A. dhufarensis, A. obesum*, and *C. austroarabica* and their interaction with the abilities to produce extracellular enzymes (phosphatase, cellulase, and glucosidase).

The results showed varying activities of the extracellular enzymes among the three studied plant species. The activities of glucosidase, cellulase, and esterase were significantly higher (*p <* 0.0001) in the rhizosphere of *A. dhufarensis* than in the rhizosphere of *C. austroarabica* and *A. obesum* (Table 3). In contrast, phosphatase activity was significantly higher (*p <* 0.001) in *A. obesum* than the other plant rhizospheres. The ACC deaminase activity was significantly (*p <* 0.0002) higher (~two-fold) in *A. dhufarensis* than in other species. However, low activity of ACC deaminase was observed in *C. austroarabica* (Table 3). The three rhizosphere soil samples from the plants showed varying concentrations of indole-3-acetic acid (IAA) content. Among the plants, the rhizosphere of *A. obesum* showed significantly higher (*p <* 0.0029) IAA content compared to the other two species (Table 3). *C. austroarabica* showed the lowest amount of IAA in the rhizosphere soil. The canonical correlation analysis (CCA) with regard to the presence of enzymes cellulase, glucosidase, and phosphatase was conducted to understand the correlation for the diversity of fungal and bacterial communities in *A. obesum*, *A. dhufarensis*, and *C. austroarabica* (Figure 6). In the case of fungal communities, CCA1 was 0.9218 and CCA2 was 0.3330, whereas in the case of bacterial communities, CCA1 was 0.7380 and CCA2 was 0.2401. A positive correlation was observed in *C. austroarabica* for the phosphatase enzyme for bacteria and fungi. A positive correlation was recorded for cellulase activity in *A. obesum* for bacteria and fungi, whereas glucosidase had a negative correlation for both bacteria and fungi (Figure 6).

## 4. Discussion

The results showed diverse niche of microorganisms in the rhizosphere of three plant species. This was also evidenced from the soil physical and chemical properties suggesting a complete segregation of the two locations i.e., dry tropical to the complete arid land system. Comparing both types of the rhizosphere from arid plant species could be essential to understand the major microbial associations. Although, the majority of the present insights into the interactions and processes of rhizosphere microbiome have come from studies on model plants such as *Arabidopsis thaliana* and *Medicago truncatula* and agricultural or horticultural crops [10,26,49], nonetheless, a reasonable progress has also being made in elucidating the microbial ecology of non-cultivated plant species [16,17,26]. Some studies also showed that how microbial associations impact the resource allocation, biodiversity and above-ground interactions with herbivores and their natural enemies [50,51]. Understanding microbial diversity across different soil types and locations of wild plants could also help in future expansion of agricultural activities in broader ecological niches and wastelands.

To some extent, microbial players and their abundances depends not only on the biogeography of the host plant species but also on host genotype, which is still being investigated by comparing microbial communities of the sample plant during varying seasonal conditions [52]. The present study elucidated the fungal and bacterial association of three medicinal plant species that displayed a varying response in the metagenomics data output as well as the number of OTUs. This finding was also validated in the recent studies that demonstrated host-specific characteristics such as a wide variety of morphology [53] and genomics [54,55] could convincingly affect the microbiome structure and diversity [16,56]. Although the climatic, soil, and plant growth parameters were quite similar, *A. dhufarensis*, *C. austroarabica* and *A. obesum* possess considerably different features in their growth, morphology, and genetic makeup, resulting in a varying nature of bacterial and fungal communities in the rhizosphere. This substantial difference in the microbial diversity can be attributed to the microsite niche heterogeneity [57,58].

The roots and their exudates can reduce the niche heterogeneity, which in turn affects the diversity and abundance of fungal and/or bacterial communities [7,59]. *A. dhufarensis* and *A. obesum* are known as sap-producing plants [37,60] in their phyllosphere continuum, which naturally becomes part of the rhizosphere either by root exudation or by wounding through herbivory. *C. austroarabica*, and is also rich in the essential oils [59]. Such host plant potentials can also result in the distribution and occurrence of certain classes of microbial communities. Therefore, a varying composition of OTUs was observed for the three medicinal plants. A similar conclusion was drawn when root exudates of maize and soybean shown drastic effects in the rhizosphere bacterial community structure and composition [61]. Rasmann and Turlings (2016) [62] recently suggested that the plant kind and its root exudation could influence the mutualistic interaction in the rhizosphere. In addition, the immediate changes in the soil attributes (pH, water, and C availability) either climatically or by the host itself and it can increase or reduce the abundance of rhizosphere microbiomes [27,50]. In addition, the difference in microbial communities associated with *A. dhufarensis* suggests that the microbiome of a species or cultivar exhibits both specific microbial lineages with host-specific abundance patterns and a conserved core microbiome [57,58].

In addition to the abundance, the distribution of microbial communities also differed across the three plant species. Although *Basidiomycota* and *Ascomycota* were abundant phyla, the contribution of unidentified fungi was still high in the three plants. This suggests the presence of novel fungal diversity in rhizosphere that have yet to be described. This report is consistent with previous studies on semi-arid land plants [27,34,36]. *Corynascus*, which has been classified as thermophilic in arid land ecosystems, was abundant in *C. austroarabica,* suggesting its dominant role in countering climatic perturbations. In addition, *Corynascus kuwaitiensis, *Cochliobolus** sp. and *Ceratobasidium* sp. were also abundant in the three rhizosphere samples. Previously, these were also found in the root zones of date palms [63,64], agave [27] and grasses [64,65] that are widely grown in arid land ecosystem.

In case of bacterial communities, *Acidobacteria, Actinobacteria*, *Bacteroidetes,* and *Proteobacteria* were highly abundant bacterial phyla. These are a few of the dominant bacterial species found in metagenomic dataset obtained from various plants and rhizospheres [57]. Similarly, increased abundance of *Proteobacteria* and decreased presence of *Acidobacteria* in the plant-rhizosphere samples with respect to different hosts were previously found with *Agave* species [27], suggesting a major community structure associated with the arid land plants. These have also been reported in some of the important medicinal plants, such as *Panax ginseng* [66], *Thymus zygis* [36], *Polygonum cuspidatum* [33], *Rhododendron arboretum* [35], *Sapindus saponaria* [53], *Taxus baccata* and *Aloe vera* [34,67]. Nonetheless, distribution of phyla including *Chloroflexi, Planctomycetes,* and *Firmicutes* in *C. austroarabica* rhizosphere and *Cyanobacteria* in *A. obesum* rhizosphere were significantly different, suggesting host-specific microbe management as indicated by Berendsen et al. [17]. The presence of a considerably higher number of “unidentified” sequences in bacteria might be due to (i) presence of a large number of sequences of uncultured microbes, (ii) presence of less sequenced microbial genomes, and/or (iii) absence of related orthologous nucleotide sequences in NCBI [16,68]. Since these plant species have been analyzed for the first time, unidentified sequences could not be associated with the potential survival of these three plants in harsh environmental conditions.

The holobiont (plants and their microbiota) plays a collective role in intergenic function and development of ecological niche. In this reciprocal interaction, production of bioactive metabolites, including extracellular enzymes and phytohormones, can subsequently pave the way for viable growth of the hosts [15,69]. These extracellular enzymes target various macromolecules, such as carbohydrates, lignin, organic phosphate, proteins, and sugar-based polymers, for their degradation into transportable products throughout the cells and to continue heterotopic metabolism [70]. In addition to establishing an association with host, these enzymes also initiate the action of extracellular hydrolysis to counteract plant pathogenic infection [14]. We found considerable higher concentrations of cellulases, glucosidases, esterase, and ACC deaminase in the rhizosphere samples of *A. dhufarensis*. However, phosphatase and IAA were high in *A. obesum*. Cellulase allows the bioconversion of cellulose and its modification into simple carbohydrates that act as carbon source for microbes [71]. The glucosidase enzyme hydrolyses starch and glycogen and converts them into monomers of carbohydrates [72]. Along with cellulase, glucosidase also plays an important role in providing carbon sources for the plants. Similarly, phosphatase degrades phosphoric acid monoesters into phosphate ions and alcohol [73]. Phosphate is one of the most important macronutrients of plant and plays diverse roles in plant growth and development, including root development and colonization of rhizospheric microbes [4,74,75]. Therefore, the presence of cellulase, glucosidase, and phosphatase in rhizosphere possess considerable significance. These enzymes, along with IAA production by associated microflora, have a high impact on the plant health and fitness against abiotic stresses [15,48]. In addition, higher ACC deaminase in *A. dhufarensis* can be attributed to abundance of *Bacteroidetes,* which have been shown extensively to produce ACC deaminase in rhizosphere [76,77]. IAA, on the other hand, was high in the rhizosphere of *A. obesum,* which could contribute to the abundance of *Actinobacteria*. *Actinobacteria* are known to produce IAA, as previous studies have shown [15,78]. This could also be attributed to survivability potentials of these three plant species during low water and nutrient availability.

In conclusion, the current results provide a genomic basis to enhance our understanding of these complex and dynamic microbial interactions with plants of sub-tropical arid ecosystems. Overall, our results are in parallel with recent metagenomic data on diversity of microbiomes associated with dry tropical to arid land plants. These plant species were studied for the first time. The identification of specific taxa, particularly at species level, can provide a new insight for future research on the associated functions and reciprocation of enriched species in rhizosphere of these medicinally important plants. Furthermore, we also assume that the secretion of exozymes and essential metabolites at microbial community level can enhance the ability of these plant species to withstand harsh sub-tropical environmental conditions.

## Figures and Tables

**Table 1:** Physio-Chemical attributes of soils collected from the different rhizospheres of three plants.

Parameters	*A. obesum*	*A. dhufarensis*	*C. austroarabica*
Temperature (°C)	37	37.3	41.3
Moisture contents (%)	18.7	19	10.1
Clay (%)	8.1 ± 1.01a	6.6 ± 0.9b	2.4 ± 0.4c
Sand (%)	34 ± 2.1c	38.5 ± 2.1a	78.3 ± 0.6a
Silt (%)	19.6 ± 1.01a	17.6 ± 0.8a	11.8. ± 1.5b
Gravel (%)	38.3 ± 1.01a	37.3 ± 0.8a	2.5. ± 0.3b
Bulk density (%)	4.7 ± 0.8a	3.9 ± 0.7a	1.1 ± 0.4b
Organic matter (%)	4.2 ± 0.6a	5.2 ± 0.4a	0.9 ± 0.1b
Texture	sandy loam	sandy loam	sandy
EC (dSm^−1^)	31 ± 1.4a	30 ± 1.9a	2.1 ± 0.7b
pH	7.3 ± 0.8a	7.6 ± 0.4a	6.8 ± 0.4b
Nitrates (mg/kg)	3.9 ± 0.4a	3.4 ± 0.7a	0.8 ± 0.2b
Phosphorus(mg/kg)	2.1 ± 0.3a	2.2 ± 0.4a	0.4 ± 0.1b

Values in each column are the mean of five replications and presented with standard error. The different letter in each row showed that the values are significantly different (*p* < 0.05).

**Table 2:** MiSeq sequencing and bacterial diversity estimators of the rhizosphere of three medicinally important plants.

Plant	Replicate	Total No. of Sequences	No. of OTUs *	Chao1	Shannon
Fungal communities					
*A. dhufarensis*	1	69673	131	58.5	1.9
	2	48156	104	47.3	1.7
	3	26999	85	45.2	2.2
*A. obesum*	1	65158	176	88.0	2.7
	2	51709	196	81.5	3.0
	3	78490	229	108.0	3.0
*C. austroarabica*	1	42156	50	43	2.4
	2	39003	49	44	2.6
	3	33497	63	41	2.7
Bacterial communities					
*A. dhufarensis*	1	150605	3966	1768.5	5.7
	2	133210	3241	1690.5	5.7
	3	126210	4834	1694.7	6.0
*A. obesum*	1	206761	3622	1754.2	5.5
	2	176014	3296	1748.8	5.3
	3	210956	3717	1788.0	5.5
*C. austroarabica*	1	152820	2812	1232.4	6.0
	2	151785	2854	1232.7	6.0
	3	149156	4616	1375.7	6.1

* Operational taxonomic unit at 3% sequence dissimilarity based on equal subsets of sequences for all samples, Chao1 is based on rare OTUs in a given sample and Shannon abundance-based coverage.

**Table 3:** Exozymes and essential metabolites of the rhizosphere soil of the three plants.

Assays	Enzyme Properties			Plant Species		
	Function	Substrate	Conc. (μM)	*A. dhufarensis*	*A. obesum*	*C. austroarabica*
Cellulase (1,4- β –cellobiosidase; (μmol h^−1^ g^−1^)	Cellulose to disaccharide	4-MUB-phosphate 3.1.3.1	10–100	157.1 ± 1.2 ^a^	134.2 ± 1.0 ^b^	105.8 ± 1.0 ^c^
Phosphatase (μmol h^−1^ g^−1^)	Phosphomonoesters to phosphate	4-MUB-β-D-cellobioside 3.2.1.91	10–100	9.6 ± 0.2 ^b^	11.4 ± 0.9 ^a^	5.2 ± 0.6 ^c^
Glucosidase (β-1,4-glucosidase; (μmol h^−1^ g^−1^)	Cellulose to glucose	4-MUB-β-D-glucopyranoside 3.2.1.21	10–100	16.9 ± 0.8 ^a^	13.8 ± 0.4 ^b^	5.5 ± 0.7 ^c^
Esterase (μmol h^−1^ g^−1^)	Acid to alcohol (hydrolyses)	4-Methylumbelliferyl butyrate	10–100	152.0 ± 1.01 ^a^	131.1 ± 2.0 ^b^	117.1 ± 2.7 ^c^
IAA (µmol/mL)	Plant root development	-	100	129.5 ± 2.8 ^b^	159.8 ± 2.9 ^a^	122.6 ± 3.4 ^bc^
ACC deaminase (nmol α-ketobutyrate mg^−1^ h^−1^)	Lowering plant ethylene levels	ACC	100	181.2 ± 2.8 ^a^	98.1 ± 2.8 ^b^	39.8 ± 2.8 ^c^

The different letters in each row for each parameter shows a significant difference (*p* < 0.05) as evaluated by Duncan’s multiple range test (SAS v9.0, CA, USA). *Adenium obesum*, *Aloe dhufarensis* and *Cleome austroarabica.*

## Supplementary Materials

The following are available online at https://www.mdpi.com/2076-2607/8/2/213/s1, Table S1. MiSeq output summary for ITS and 16S rDNA of the rhizosphere region of the plant species. Table S2. Merging pre-processing & clustering of the data obtained from MiSeq data and the associated quality parameters. Table S3. Two-way ANOVA of the diversity of the ITS and 16S rDNA datasets. Table S4 Fungal OTUs showing the shared taxonomic assignments of *A. dhufarensis, A. obesum* and *C. austroarabica.* Table S5. Distribution of various phyla in the bacterial communities in the rhizosphere of *A. dhufarensis, A. obesum* and *C. austroarabica.* Table S6. Distribution of various species in the bacterial communities in the rhizosphere of *A. dhufarensis, A. obesum* and *C. austroarabica.* Figure S1. Calculated rarefaction curves of the observed OTUs (sequences that possess above 97% similarity are defined as one OTU) richness in rhizosphere soil samples of *A. dhufarensis, A. obesum* and *C. austroarabica.* Figure S2. The principal coordinate analyses (PCoA) of pairwise Bray-Curtis distance matrixes of filtered OTU tables for the fungal and bacterial communities in the rhizosphere of *A. obesum*, *A. dhufarensis* and *C. austroarabica* plant species.
