# Supplementary material for: Rhizosphere Microbiome of Arid Land Medicinal Plants and Extra Cellular Enzymes Contribute to Their Abundance
Source: Microorganisms. 2020 Feb 5;8(2):213. doi: 10.3390/microorganisms8020213 (PMC7074696; doi:10.3390/microorganisms8020213)
Supplement: Supplementary file 1 [file microorganisms-08-00213-s001.zip › microorganisms-664070-supplementary-for publication/Table S1.docx]

**Table S1**: MiSeq output summary for ITS and 16S rDNA of rhizospheric region of plant species

| **Parameters** | **16S** | **ITS** |
| --- | --- | --- |
| Total data output | 786Mb | 361.4Mb |
| Average bases | 546,307 | 1,219,536 |
| Mean read counts | 32,135 | 71,737 |
| Maximum read counts | 43,122 | 139,360 |
| Chimera reads | 489,304 | 21,820 |
| Low quality | 148,653 | 178,399 |
| Others | 2,059,305 | 437,011 |
