# Supplementary material for: Rhizosphere Microbiome of Arid Land Medicinal Plants and Extra Cellular Enzymes Contribute to Their Abundance
Source: Microorganisms. 2020 Feb 5;8(2):213. doi: 10.3390/microorganisms8020213 (PMC7074696; doi:10.3390/microorganisms8020213)
Supplement: Supplementary file 1 [file microorganisms-08-00213-s001.zip › microorganisms-664070-supplementary-for publication/Table S2.docx]

**Table S2:** Merging Preprocessing & Clustering of the data obtained from MiSeq data and the associated quality parameters

| **Sample Name** | **Total Bases** | **Read Count** | **GC (%)** | **Q20 (%)** | **Q30 (%)** |
| --- | --- | --- | --- | --- | --- |
| **Fungal communities** | | | | | |
| *Aleo 1* | 64,661,056 | 177,806 | 50.51 | 98.44 | 94.34 |
| *Aleo 2* | 34,893,129 | 93,950 | 50.86 | 98.35 | 94.01 |
| *Aleo 3* | 41,323,534 | 118,603 | 55.49 | 98.54 | 94.3 |
| *Adenium1* | 34,606,310 | 94,402 | 52.66 | 98.29 | 93.73 |
| *Adenium 2* | 32,663,102 | 91,309 | 53.44 | 98.42 | 94.01 |
| *Adenium 3* | 44,510,549 | 120,474 | 52.68 | 98.29 | 93.79 |
| *Cleome1* | 35,074,961 | 95,537 | 50.41 | 98.29 | 93.74 |
| *Cleome 2* | 34,379,399 | 94,951 | 51.68 | 98.35 | 93.9 |
| *Cleome 3* | 39,333,014 | 109,282 | 53.48 | 98.21 | 93.25 |
| **Bacterial communities** | | | | | |
| *Aleo 1* | 92,206,986 | 205,063 | 56.12 | 97.79 | 92.92 |
| *Aleo 2* | 80,472,929 | 178,985 | 56.59 | 97.65 | 92.41 |
| *Aleo 3* | 78,431,826 | 173,942 | 58.08 | 97.6 | 92.28 |
| *Adenium 1* | 92,306,905 | 206,761 | 57.15 | 97.74 | 92.64 |
| *Adenium 2* | 78,616,337 | 176,014 | 57.23 | 97.76 | 92.69 |
| *Adenium 3* | 94,299,035 | 210,956 | 57.14 | 97.71 | 92.6 |
| *Cleome1* | 92,598,930 | 206,216 | 57.97 | 97.67 | 92.39 |
| *Cleome 2* | 89,436,982 | 199,271 | 57.76 | 97.74 | 92.58 |
| *Cleome 3* | 88,426,366 | 197,137 | 57.83 | 97.7 | 92.42 |
