# Supplementary material for: Rhizosphere Microbiome of Arid Land Medicinal Plants and Extra Cellular Enzymes Contribute to Their Abundance
Source: Microorganisms. 2020 Feb 5;8(2):213. doi: 10.3390/microorganisms8020213 (PMC7074696; doi:10.3390/microorganisms8020213)
Supplement: Supplementary file 1 [file microorganisms-08-00213-s001.zip › microorganisms-664070-supplementary-for publication/Table S3.docx]

**Table S3; Two-way ANOVA analysis of the diversity of the ITS and 16S rDNA dataset**

| **Parameters** | **Microbial communities** | | **Plant species** | | **Microbes x plant species** | |
| --- | --- | --- | --- | --- | --- | --- |
|  | **% Variation** | **P value** | **% Variation** | **P value** | **% Variation** | **P Value** |
| OTU richness | 98.79 | < 0.0001 | 0.3080 | 0.0159 | 0.4528 | 0.0041 |
| Chao1 richness | 98.38 | < 0.0001 | 0.4925 | 0.0186 | 0.5340 | 0.0140 |
| Shannon diversity index | 91.59 | < 0.0001 | 2.156 | 0.0008 | 5.033 | < 0.0001 |
| Simpson diversity index | 49.18 | < 0.0001 | 11.40 | 0.1295 | 11.59 | 0.1252 |
