# Supplementary material for: Rhizosphere Microbiome of Arid Land Medicinal Plants and Extra Cellular Enzymes Contribute to Their Abundance
Source: Microorganisms. 2020 Feb 5;8(2):213. doi: 10.3390/microorganisms8020213 (PMC7074696; doi:10.3390/microorganisms8020213)
Supplement: Supplementary file 1 [file microorganisms-08-00213-s001.zip › microorganisms-664070-supplementary-for publication/Table S4.docx]

**Table S4:** Fungal OTUs showing the shared taxonomic assignments of the three plants (*A. dhufarensis, A. obessum* and *C. austroarabica*)

| **Genus** | ***Aleo*** | ***Adenium*** | ***Cleome*** |
| --- | --- | --- | --- |
| *Ceratobasidium* | 13586 | 12521 | 12 |
| *Ascotricha* | 14495 | 0 | 40 |
| *Fusarium* | 2 | 1633 | 1 |
| *Paecilomyces* | 155 | 1156 | 1 |
| *Fomitopsis* | 1 | 17 | 0 |
| *Corynascus* | 0 | 315 | 4724 |
| *Exophiala* | 1 | 0 | 0 |
| *Rhizopycnis* | 0 | 0 | 0 |
| *Stachybotrys* | 1 | 0 | 0 |
| *Alternaria* | 22 | 38 | 114 |
| *Campylocarpon* | 0 | 0 | 0 |
| *Chaetomium* | 27 | 1151 | 0 |
| *Myrothecium* | 1 | 0 | 0 |
| *Dactylella* | 1 | 2 | 0 |
| *Lecanicillium* | 0 | 728 | 0 |
| *Phanerochaete* | 0 | 0 | 0 |
| *Purpureocillium* | 0 | 178 | 0 |
| *Lophiostoma* | 0 | 482 | 1 |
| *Teratosphaeria* | 0 | 468 | 0 |
| *Camarosporium* | 0 | 103 | 0 |
| *Phoma* | 2 | 776 | 0 |
| *Penicillium* | 0 | 0 | 47 |
| *Entoloma* | 0 | 242 | 0 |
| *Lepiota* | 0 | 407 | 0 |
| *Preussia* | 0 | 124 | 0 |
| *Microascus* | 363 | 2 | 0 |
| *Phoma* | 0 | 122 | 0 |
| *Acremonium* | 65 | 5182 | 0 |
| *Cladorrhinum* | 0 | 13 | 0 |
| *Metarhizium* | 1 | 1 | 0 |
| *Stachybotrys* | 8 | 10 | 0 |
| *Scolecobasidium* | 9 | 59 | 0 |
| *Glomus* | 1 | 1 | 1 |
| *Sarcinomyces* | 0 | 103 | 0 |
| *Auxarthron* | 0 | 73 | 0 |
| *Devriesia* | 7 | 0 | 0 |
| *Chaetomium* | 0 | 199 | 0 |
| *Cochliobolus* | 0 | 217 | 0 |
| *Aspergillus* | 552 | 249 | 794 |
| *unidentified* | 28985 | 10792 | 3268 |
| *Unassigned* | 110375 | 67644 | 59685 |
| *Total* | 139365 | 78439 | 62957 |
