# Supplementary material for: Rhizosphere Microbiome of Arid Land Medicinal Plants and Extra Cellular Enzymes Contribute to Their Abundance
Source: Microorganisms. 2020 Feb 5;8(2):213. doi: 10.3390/microorganisms8020213 (PMC7074696; doi:10.3390/microorganisms8020213)
Supplement: Supplementary file 1 [file microorganisms-08-00213-s001.zip › microorganisms-664070-supplementary-for publication/Table S5.docx]

**Table S5:** Distribution and taxonomy abundance percentage of various phyla of bacterial communities in the rhizosphere of three plants (*A. dhufarensis, A. obessum* and *C. austroarabica*)

| **Phylum** | ***Aleo*** | ***Adenium*** | ***Cleome*** |
| --- | --- | --- | --- |
| *Other* | 0.29% | 0.26% | 0.26% |
| *Unassigned/Unidentified* | 3.12% | 3.56% | 7.42% |
| *Acidobacteria* | 3.74% | 2.45% | 5.48% |
| *Actinobacteria* | 27.83% | 31.21% | 20.35% |
| *Armatimonadetes* | 0.17% | 0.54% | 0.01% |
| *Bacteroidetes* | 12.74% | 5.12% | 8.81% |
| *Chlamydiae* | 0.01% | 0.00% | 0.44% |
| *Chloroflexi* | 3.09% | 2.39% | 10.34% |
| *Deinococcus-Thermus* | 0.02% | 0.15% | 0.00% |
| *Fusobacteria* | 0.19% | 0.02% | 0.23% |
| *Gemmatimonadetes* | 2.34% | 0.45% | 0.73% |
| *Planctomycetes* | 8.12% | 1.01% | 13.58% |
| *Proteobacteria* | 17.98% | 12.92% | 21.76% |
| *Verrucomicrobia* | 9.08% | 10.24% | 3.74% |
| *BRC1* | 0.01% | 0.00% | 0.00% |
| *Candidatus Saccharibacteria* | 0.57% | 0.15% | 0.57% |
| *Cyanobacteria* | 5.77% | 26.88% | 0.05% |
| *Firmicutes* | 3.10% | 0.59% | 4.21% |
| *Nitrospirae* | 0.11% | 0.07% | 0.01% |
| *Parcubacteria* | 0.01% | 0.02% | 0.02% |
| *candidate division WPS-1/WPS-2* | 0.98% | 1.43% | 0.68% |
