# Supplementary figures and images for: Rhizosphere Microbiome of Arid Land Medicinal Plants and Extra Cellular Enzymes Contribute to Their Abundance
Source: Microorganisms. 2020 Feb 5;8(2):213. doi: 10.3390/microorganisms8020213 (PMC7074696; doi:10.3390/microorganisms8020213)

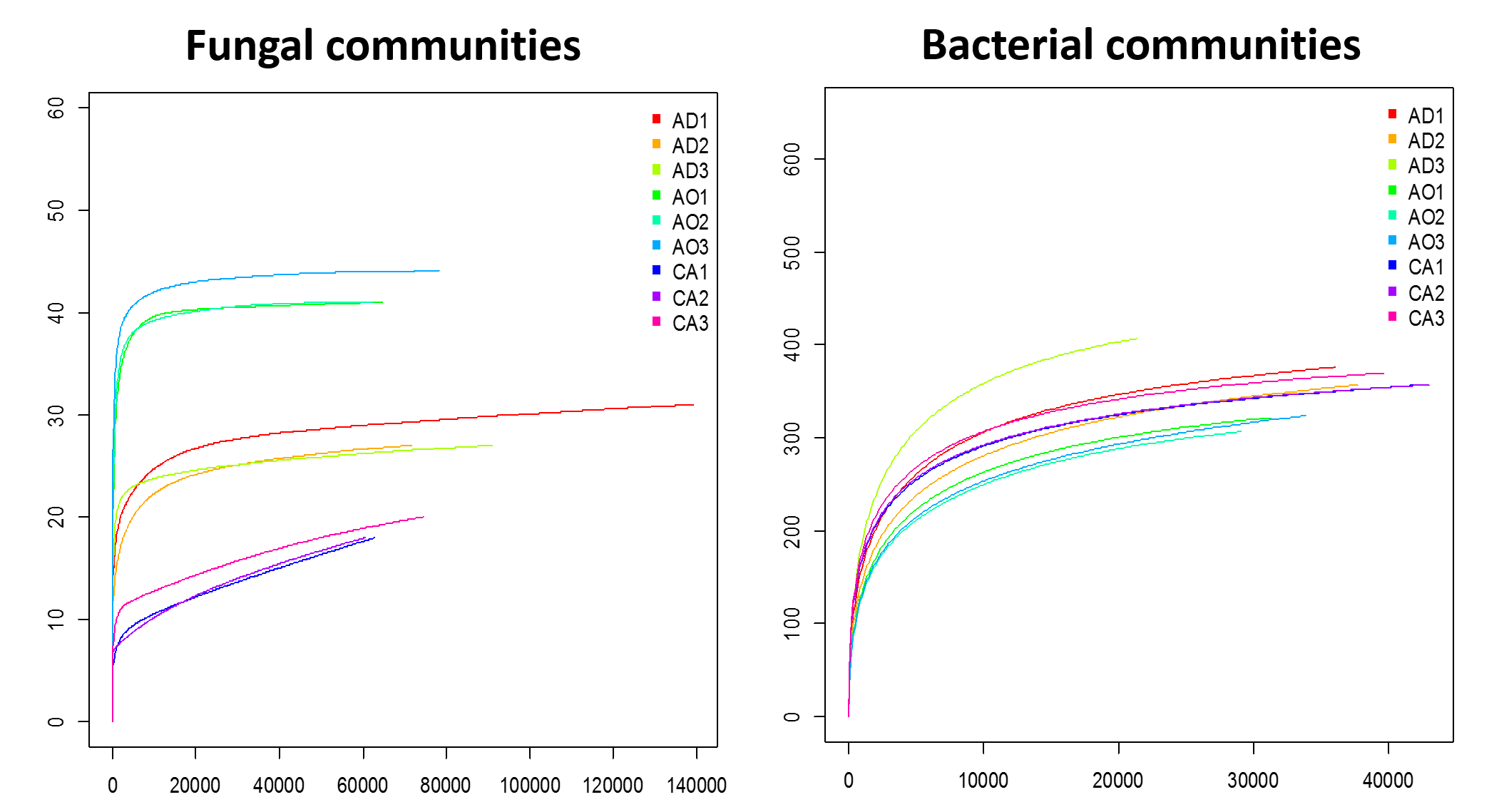

Supplement: Supplementary file 1 [file microorganisms-08-00213-s001.zip › microorganisms-664070-supplementary-for publication/Figure S1.tif]

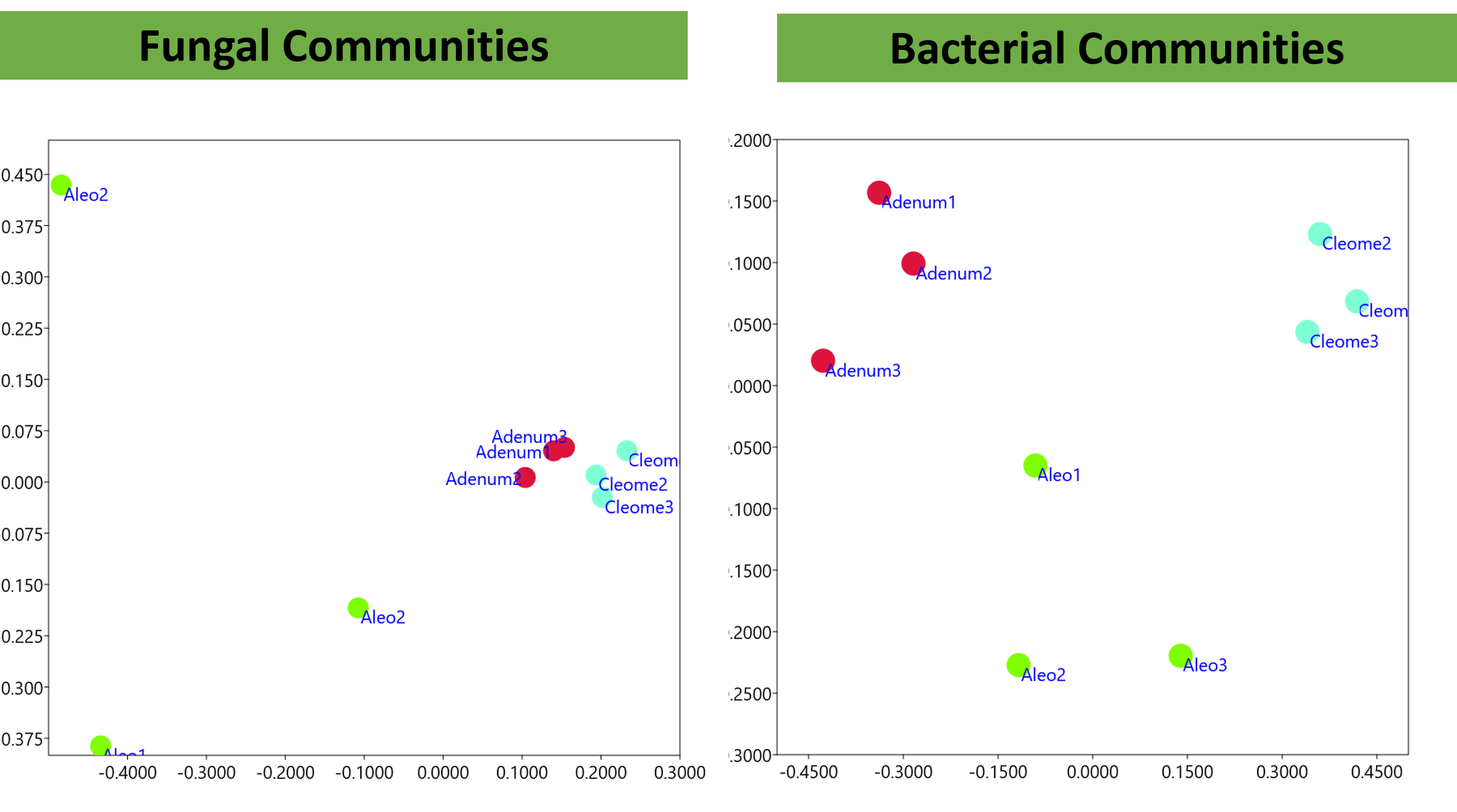

Supplement: Supplementary file 1 [file microorganisms-08-00213-s001.zip › microorganisms-664070-supplementary-for publication/Figure S2.tif]
